# Halogenation of nucleic acid structures: from chemical biology to supramolecular chemistry

**DOI:** 10.1039/d5cb00077g

**Published:** 2025-05-22

**Authors:** Catalina Nicolau, Julia Requena-Ramírez, Jorge González-García, Antonio Bauzá

**Affiliations:** a Ctra. de Valldemossa, km. 7.5, Universitat de les Illes Balears 07122 Palma de Mallorca Islas Baleares Spain antonio.bauza@uib.es; b Department of Inorganic Chemistry, Institute of Molecular Science (ICMol), University of Valencia Catedrático José Beltrán 2 46980 Paterna Spain jorge.gonzalez@uv.es

## Abstract

Halogenation of organic molecules is a widespread resource used in the fields of chemical biology and rational drug design to improve the binding affinity or solubility of a given compound. Interestingly, the incorporation of Br and I in DNA/RNA bases has been routinely carried out during decades to facilitate the structural determination of nucleic acids, without accounting for its impact in the DNA/RNA structure and molecular recognition events involving other biological entities (*e.g.* proteins and peptides). This is of critical importance, since halogens undergo non-covalent binding (specially Br and I) through the formation of halogen bonding interactions, thus structurally influencing peptide/protein–DNA/RNA binding poses or altering the supramolecular architecture of isolated nucleic acid structures. In this review, the physical nature of halogen bonds involving nucleobases as well as their implications in (i) the formation of protein–DNA/RNA complexes and (ii) the stabilization of non-canonical DNA/RNA structures will be discussed, focusing on the role of this non-covalent interaction as a promising tool in nucleic acid chemistry.

## Abstract 2

In this review article, the impact of nucleobase halogenation in chemical biology and supramolecular chemistry has been discussed, highlighting the importance of halogen bonding interactions as a novel molecular recognition tool.

## Graphical abstract

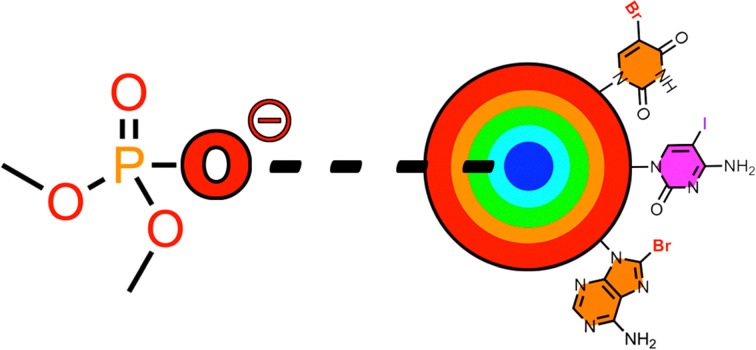

## Introduction

### Chemical modification of nucleic acids

Chemically modifying DNA and RNA bases is a crucial process in the regulation of gene expression,^1,2^ being essential for understanding the fundamental mechanisms of epigenetics.^1^ These modifications affect both the secondary nucleic acid structure and the protein–DNA/RNA binding mechanisms, thereby regulating processes related to cellular survival.^3^ During the last few stages of the past century, there was a significant increase in research related to the covalent modification of DNA/RNA, primarily due to its promising applications in clinical medicine, such as the use of biomarkers for disease prevention and diagnosis, which has led to a better understanding of the role of epigenetics in various diseases.^4–7^

**Fig. 1:**
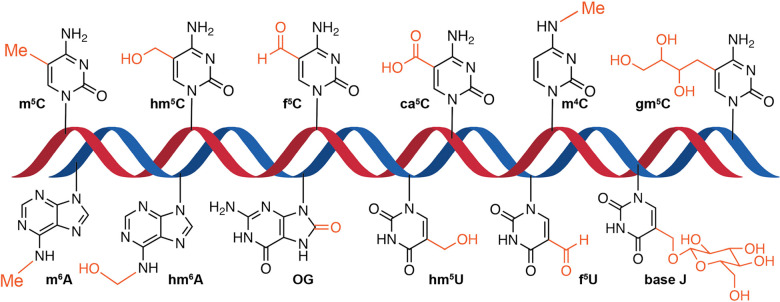
Schematic representation of modified A and C bases present in DNA. m^5^C, 5-methylcytosine; hm^5^C, 5-hydroxymethylcytosine; f^5^C, 5-formylcytosine; ca^5^C, 5-carboxylcytosine; m^4^C, N4-methylcytosine; gm^5^C, 5-glycerylmethylcytosine; m^6^A, N6-methyladenosine; hm^6^A, N6-hydroxymethyladenine; OG, 8-oxo-7,8-dihydroguanine; hm^5^U, 5-hydroxymethyluracil; f^5^U, 5-formyluracil; and base J, β-d-glucosyl-5-hydroxymethyluracil.

The chemical modification of DNA/RNA bases has been detected across all domains of life, including eukaryotic and prokaryotic organisms as well as viruses.^8^ More than 100 types of chemical modifications have been identified in RNA. Their functions are not yet clear, and they are mostly involved in tuning the translational process, controlling their stability and proper folding and determining the identity of the corresponding RNA.^9,10^ The plethora of non-canonical RNA nucleobases includes simple modifications such as methylation or acetylation in addition to hypermodified ribonucleosides containing different heterocycles, such as queuosine and wyosine.^11,12^ In contrast, there is a small fraction of DNA modifications in comparison with RNA. One notable example is 5-methylcytosine (m^5^C), discovered over 50 years ago, which remains one of the most extensively studied epigenetic marks in mammals to date.^13^ In addition to m^5^C, other canonical DNA modifications include 5-hydroxymethylcytosine (hm^5^C), 5-formylcytosine (f^5^C), 5-carboxylcytosine (ca^5^C), *N*4-methylcytosine (m^4^C), *N*6-methyladenine (m^6^A), and *N*6-hydroxymethyladenine (hm^6^A), among others (see Fig. 1).

These DNA modifications affect not only its conformation and stability but also its protein-binding mechanism. In fact, the malfunction of the epigenetic regulatory mechanisms has deep implications for human health, particularly in diseases related to (1) cancer,^14–17^ (2) obesity^18^ and diabetes,^19,20^ and (3) neurodegenerative processes.^21–24^

**Fig. 2:**
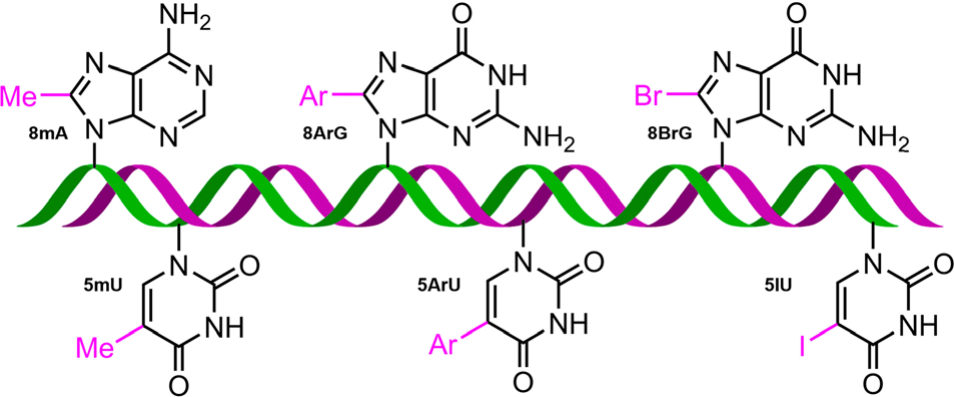
Schematic representation of non-canonically modified A, G, and U bases. 8mA, 8-methyladenine; 8ArG, 8-arylguanine; 8BrG, 8-bromoguanine; 5mU, 5-methyluracil; 5ArU, 5-aryluracil; and 5IU, 5-iodouracil.

On the other hand, the non-canonical modification of nucleic acids encompasses various transformations, such as the incorporation of methyl (–Me), halogen (–X), or aryl (–Ar) groups likely at the C8 position of purine rings^25–27^ and the C5 position of pyrimidine rings (Fig. 2),^28–30^ although N4 and N6 positions are also used. Interestingly, these modifications play a key role in the formation of alternative DNA and RNA structures.^31,32^ For instance, modifications at the C8 position of the purine ring are often used as inducting agents of *Z*-conformation and guanine quadruplex formation in both DNA and RNA. These modifications have also been employed to study alternative structures, including H-DNA and DNA triple helices,^33^ such as methylation in tRNA,^34^ which led to the formation of Levitt base pairs instead of the canonical Watson–Crick base pairs.

**Fig. 3:**
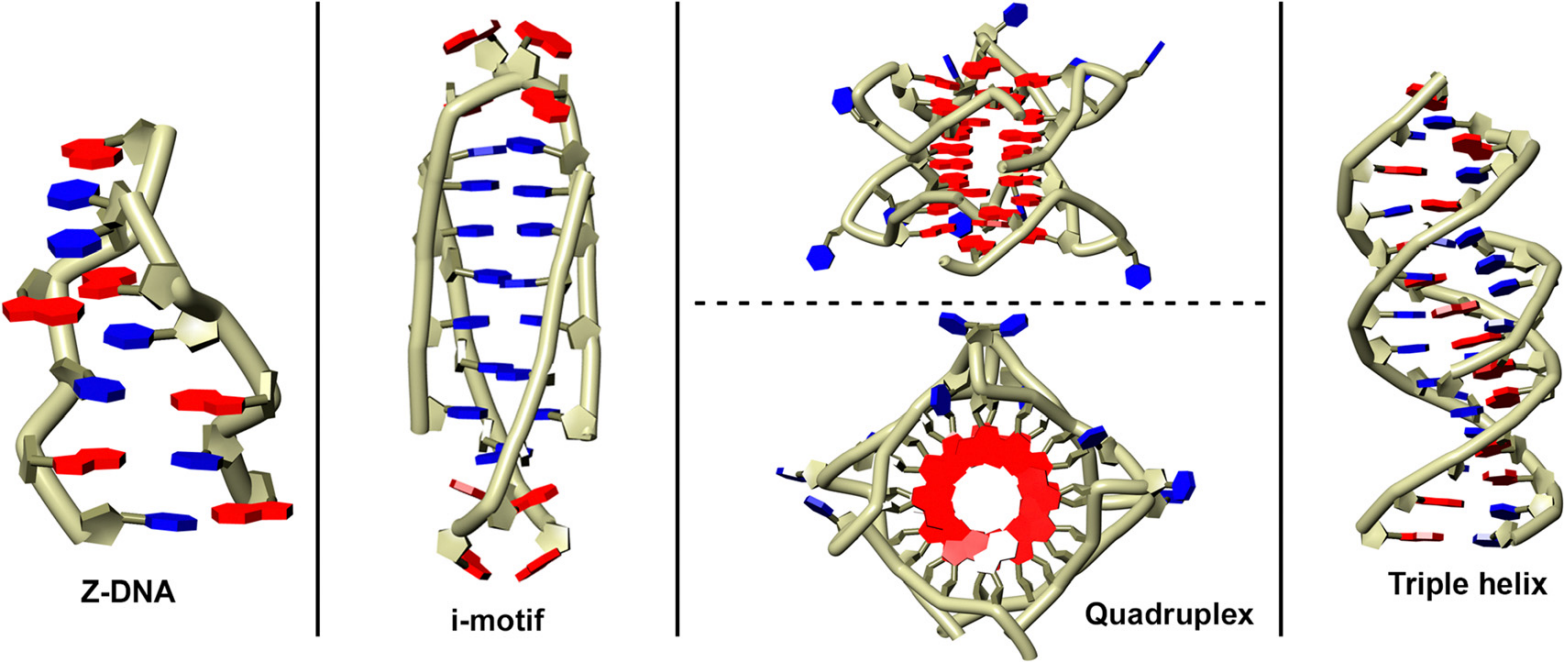
Schematic representation of several non-canonical DNA structures: Z-DNA (a), i-motif (b), G-quadruplex (c), and triple helix (d).

The conformation of ribose rings (in both DNA and RNA) is also crucial in determining the adoption of a particular nucleic acid structure (see Fig. 3). An example of this can be observed in purines, where conformational equilibrium exists between the *anti* and *syn* conformations of the ribose rings. Under physiological conditions, the typical duplex DNA conformation is dominated by the B-form, where A⋯T and G⋯C base pairs^35^ preferentially adopt the *anti* conformation. However, several studies have demonstrated that the introduction of bulky substituents at the C8 position of the purine ring shifts is enough to shift the equilibrium towards the *syn* conformation,^36,37^ thereby promoting the transition to alternative DNA structures such as Z-DNA^38–40^ or guanine quadruplexes.^41–43^ Additionally, the incorporation of halogens at the C2′ position of the ribose ring has been studied for its role in stabilizing *i*-motif DNA structures. This phenomenon is correlated with genes that predispose individuals to certain diseases and plays a vital role in DNA's biological functions, as Z-DNA sequences are often located near transcription factor binding sites.^44,45^

Despite the growing number of studies in this field, the role and conformational properties of non-canonical DNA structures are not yet fully understood. Advancing our knowledge of the effects of such nucleotide and oligonucleotide modifications is therefore crucial for gaining a deeper understanding of their structural implications for nucleic acid stability and protein–DNA binding mechanisms. To achieve this, both experimental techniques^46,47^ (such as mass spectrometry^48,49^ and DNA sequencing studies)^50^ and computational tools (primarily molecular dynamics simulations and docking studies)^51^ are employed.

In this context, ongoing efforts to develop novel theoretical tools for studying and characterizing DNA and other biomolecules have established molecular dynamics simulations as a highly valuable tool for nucleic acid analysis. Furthermore, the combination of molecular dynamics and quantum mechanics calculations enables precise insights into the structure and dynamics of large biological systems (such as protein–DNA complexes), which is crucial for deciphering, at an atomistic level, the molecular mechanisms governing nucleic acid modification and recognition.

### Nucleic acid halogenation and DNA damage

The presence of mutations in DNA is associated with genomic instability and, consequently, an increased predisposition to cancer.^52–55^ Concretely, the accumulation of DNA damage over time through these mutations acts as a trigger for uncontrolled cell proliferation and dysfunctional cellular migration mechanisms, both of which are hallmarks of cancer cells.^56,57^

There is a well-established correlation between mutations and DNA damage, with inflammatory processes being a major cause of mutagenic damage to DNA. A key feature of inflammatory responses is the production of chemical species designed to neutralize pathogenic agents. While these reactive species are essential for protecting the body against infection, they can also cause DNA damage. As a result, DNA repair mechanisms play a critical role in preventing mutations in the genetic code. However, these mechanisms may fail or become overwhelmed by excessive damage, allowing mutations to persist. Since cancer develops through the accumulation of mutations, it is concluded that unrepaired DNA damage due to inflammation contributes to cancer progression by increasing mutagenesis.

**Fig. 4:**
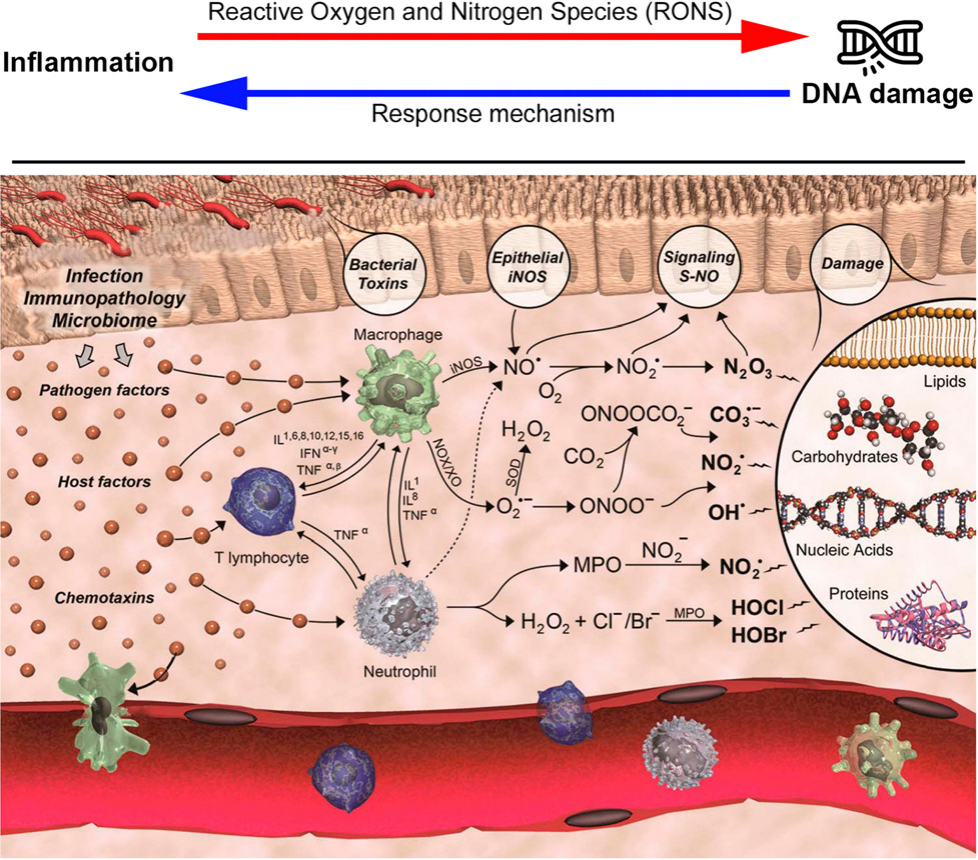
Schematic representation of the relationship between inflammation and DNA damage, as well as its contribution to cancer (top). The biological chemistry of cancer, adapted from Mangerich and collaborators (bottom).^69^

Inflammation and genomic instability are closely interconnected (Fig. 4, top), as inflammation promotes mutagenesis through the production of reactive oxygen and nitrogen species (RONS), which can directly damage DNA. In a parallel way, DNA damage can further exacerbate inflammation, creating a self-perpetuating feedback loop being regulated by a plethora of DNA repair mechanisms, transcription factors, and cellular signaling pathways. Due to the complexity of the molecular mechanisms involved in inflammation, DNA damage, and repair, these processes can become dysregulated relatively easily, ultimately leading to cancer.

In this context, various molecules have been classified as RONS, including nitric oxide (NO), which serves as a crucial cellular signaling molecule.^58–62^ Additionally, neutrophils and macrophages produce superoxide anions (O_2_^−^), while other enzymatic pathways contribute to a cascade of chemical reactions leading to the generation of a diverse array of RONS (Fig. 4, bottom). These include radicals (hydroxyl radical OH˙ and nitrogen dioxide radical NO_2_˙), anions (peroxynitrite ONOO^−^ and nitrosoperoxocarbonate ONOOCO_2_^−^), anhydrides (nitrous anhydride N_2_O_3_), hypohalogenous acids (hypochlorous acid HOCl and hypobromous acid HOBr), and hydrogen peroxide (H_2_O_2_).^63–65^ Finally, studies have demonstrated that, in addition to RONS produced by immune cells, pro-inflammatory cytokines can also stimulate intracellular RONS production.^66–68^

**Fig. 5:**
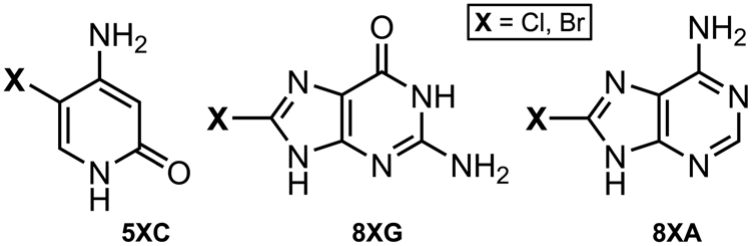
Schematic representation of the main DNA halogenation products.

The mutagenic character of halogenated DNA is associated with the generation of hypohalous acids by inflammatory cells. Specifically, neutrophils secrete the enzyme myeloperoxidase, which in turn produces hypochlorous acid (HOCl).^70–72^ On the other hand, eosinophils secrete eosinophil peroxidase, generating hypobromous acid (HOBr).^73,74^ Both molecules react with DNA during inflammatory processes, leading to the formation of halogenated products such as 5-halocytosine (5-haloC), 8-haloguanine (8-haloG), and 8-haloadenine (8-haloA) (Fig. 5).^75–78^

Among these halogenated products, the most abundant halogenated base is 5-chlorocytosine (5ClC),^79,80^ which has been detected at significantly higher levels than other DNA lesions resulting from oxidation, deamination, or peroxidation, as demonstrated in *in vivo* studies.^81,82^ Due to its substantial and persistent accumulation in tissues under inflammatory stress—possibly due to inefficient repair mechanisms—as well as in cancers associated with inflammatory pathways,^83^ 5ClC has been designated as a biomarker of chronic inflammation.^84^

### Incorporation of halogenated bases into DNA and RNA

Several studies have analyzed the effect of halogenation on the stability of base pairs in DNA and RNA, both theoretically and experimentally,^85–100^ partly due to its application as radiosensitizing agents in cancer therapies.^101,102^ In the following sections of this review, several selected examples are discussed in more detail.

### Evaluating the stability of halogenated base pairs

**Fig. 6:**
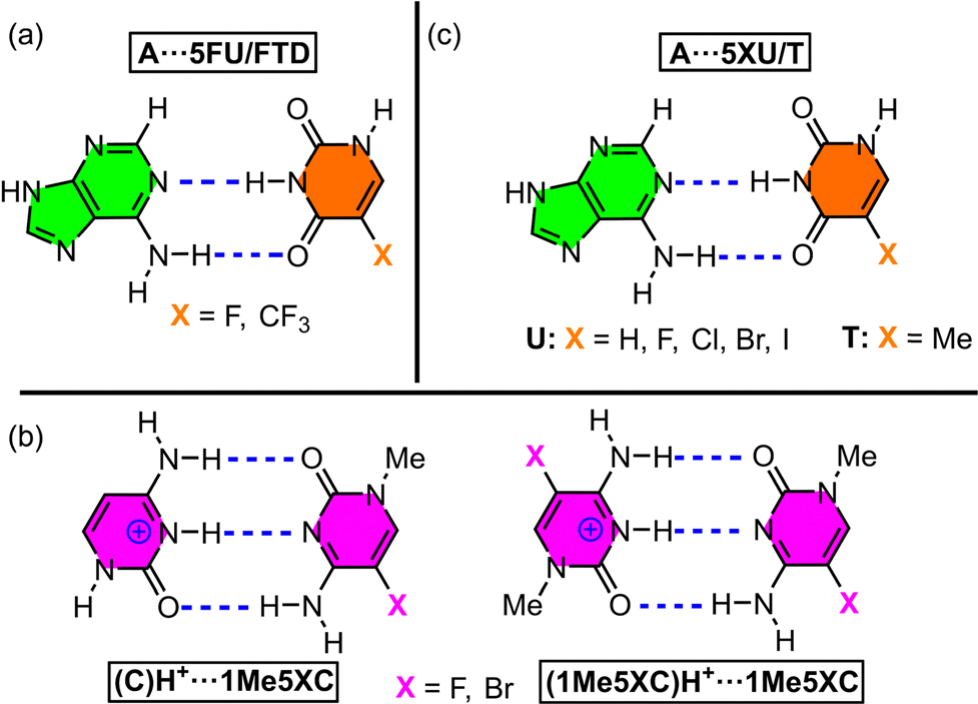
Schematic representation of the base pairs used in the studies by Koseki^85^ (a), Yang^86^ (b), and Heshmati^87^ (c).

The first selected example involves the study by Koseki and collaborators,^85^ where they performed quantum mechanics calculations and molecular dynamics simulations of nucleic acids modified with 5-trifluorothymine (FTD) and 5-fluorouracil (5FU). These modified nucleotides, particularly FTD, had previously been used as antitumor agents;^103–105^ however, their structural effects on a DNA strand had not been thoroughly investigated. To address this, theoretical calculations (MP2/6-31++G**) were performed to analyze the stability of A⋯FTD and A⋯5FU base pairs (Fig. 6a).

The results were compared with the canonical, non-halogenated base pairs A⋯T and A⋯U, revealing a slight increase in stability due to the electron-accepting effects of the –F and –CF_3_ substituents. Subsequently, molecular dynamics simulations were carried out on a DNA fragment to assess the stability and structural impact of incorporating halogenated nitrogenous bases. The authors concluded that the antitumor activity exhibited by 5-trifluoromethyl-thymine incorporation was due to the destabilizing effect of the –CF_3_ group, which was involved in the formation of intra-strand hydrogen and halogen bonds. Finally, experimental analyses were conducted using UV/visible spectroscopy, along with *in vivo* assays, to complement and corroborate the data obtained from the simulations.

Another relevant study was conducted by Yang and coworkers,^86^ where they evaluated the stability of cytosine (C)H^+^⋯C base pairs incorporating fluorine (F) and bromine (Br) atoms, and these modifications were involved in the transition from B-DNA to the *i*-motif structure. The authors employed the threshold collision induced dissociation technique using tandem ion beam mass spectrometry to study the formation of cytosine homodimers and heterodimers (Fig. 6b).

The authors concluded that halogenation at the C5 position reduced the strength of the interactions between the studied base pairs.^106,107^ This effect was significantly more pronounced in the stability of base pairs that incorporated a methylated cytosine at the C1 position. The authors suggested that the *i*-motif formation could be destabilized through C5 halogenation; however, these modified base pairs still exhibited greater stability than their neutral nitrogenous base counterparts, thus suggesting that halogenation is not sufficient to prevent *i*-motif formation but can alter the number of nucleotides required to induce its transition from a canonical Watson–Crick helix.

Another example is the work of Heshmati and collaborators,^87^ who conducted computational analyses (B3LYP/6-311G* level of theory) to assess the stability of the A⋯5FT base pair (Fig. 6c). Their aim was to explore the potential use of halogenated thymine derivatives as DNA radiosensitizers in anticancer therapy^108–110^ and observed that halogen atom incorporation led to substantial alterations in both the geometry and charge distribution of the thymine molecule, consequently affecting the stability of the A⋯5FT base pair and eventually provoking base pair opening.

**Fig. 7:**
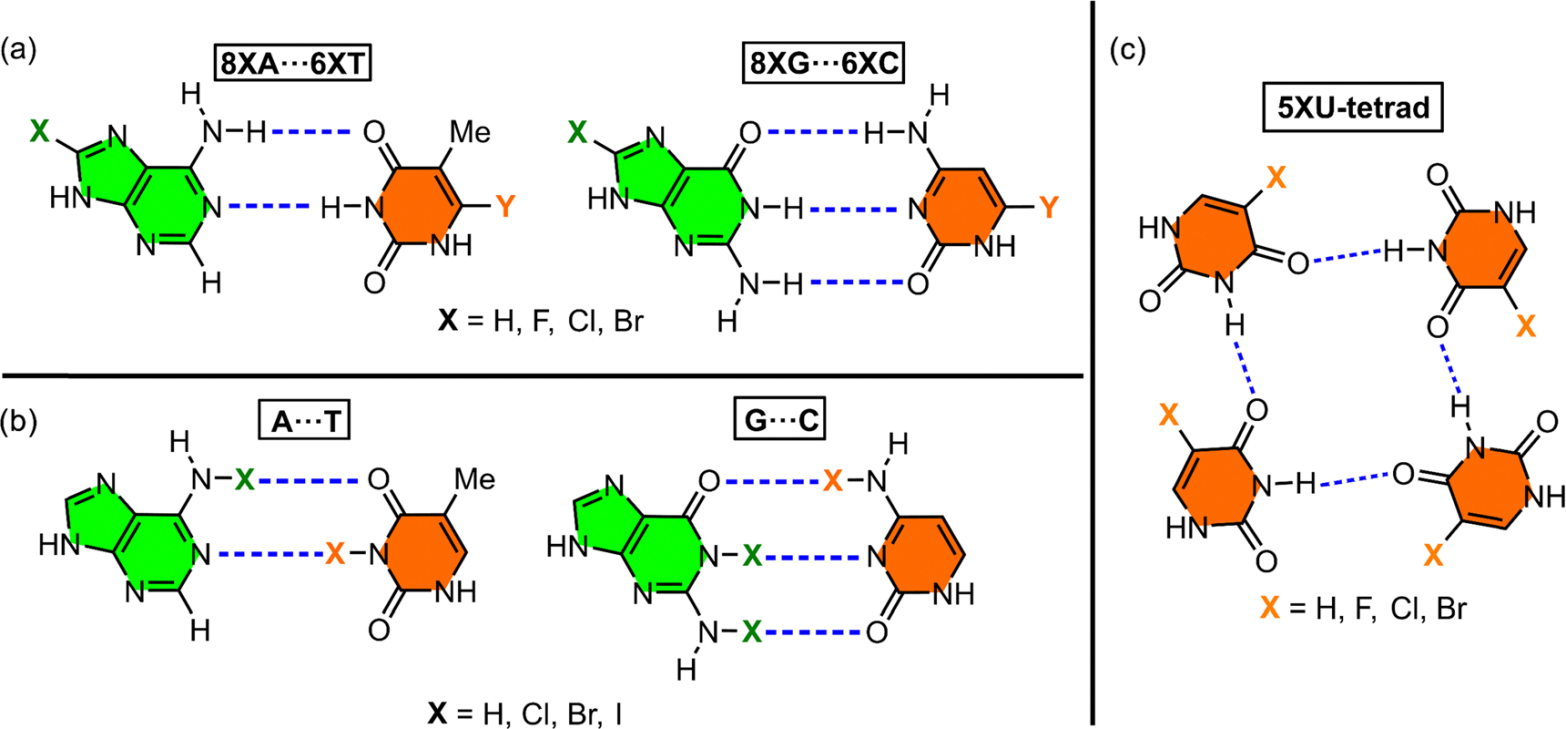
Schematic representation of the base pairs used in the studies by Guerra^91^ (a), Parker^92^ (b), and Zhao^88^ (c).

Other interesting studies encompass those conducted by Guerra^91^ and Parker^92^ and collaborators, where the stability of A⋯T and G⋯C base pairs incorporating halogenated nucleotides was analyzed (Fig. 7a and b). Concretely, Guerra and collaborators^91^ carried out theoretical evaluations (BP86/TZVP level of theory) to understand the stability of the base pairs 8XA⋯6XT and 8XG⋯6XC (X = H, F, Cl, and Br), in which the hydrogen atom at positions 8 (A, G) and 6 (T, C) was replaced by halogens (Fig. 7a). The authors concluded that the effects varied significantly depending on both the halogenation position and the base pair under study. They observed contrasting effects, such as an enhancement in hydrogen bond donor capacity (N–H) alongside a reduction in hydrogen bond acceptor capacity (O, N). In general, fluorine incorporation into the pyrimidine base reinforced base pair stability, whereas its incorporation into the purine base had the opposite effect. Additionally, the authors highlighted the significant role of orbital interactions as an additional stabilization source beyond electrostatic contributions in these halogenated base pairs.

A final example worth mentioning is the study by Zhao and coworkers,^88^ who investigated the stability of halogenated uracil tetrads (Fig. 7c) using DFT calculations (B3LYP/6-31+G** level of theory). Their results showed that stabilization energy of this ternary complex followed the order H > F > Cl > Br. Furthermore, electrostatic potential maps (MEP) revealed that the typical cation-binding site (Na^+^/K^+^) located at the center of the tetrad disappeared upon incorporation of the 5XU bases. Instead, an electron-deficient region emerged, favoring interactions with electron-rich species.

Beyond their role in modulating base pair stability through their electron-acceptor properties, halogen atoms have also been explored as substituents for conventional hydrogen bonding between bases. Specifically, Parker and collaborators^92^ performed computational studies (B3LYP/6-31G* level of theory) to assess the potential of halogen atoms as direct stabilizers of base pairs through halogen bond formation. They examined the stability of Watson–Crick base pairs G⋯C and A⋯T, in which Cl, Br, and I replaced hydrogen atoms (Fig. 7b), and compared their stability with non-halogenated base pairs. The base pairs stabilized through halogen bonding exhibited greater stability, following the trend Cl < Br < I. Among these, bromine emerged as the ideal halogen bond donor, offering a higher polarizability than chlorine and a smaller atomic volume than iodine.

### Halogen bonds in DNA and RNA supramolecular assemblies

Currently, halogen bonding (a non-covalent attractive interaction between a halogen and a Lewis base)^111,112^ has been established as a valuable molecular tool in various areas of modern chemistry, including solid-state chemistry, materials science, and supramolecular chemistry.^113^ In the field of chemical biology, halogen bonding has been mostly investigated in protein–ligand complex formation, becoming an essential tool in the design of novel therapeutic agents within pharmaceutical chemistry.^114–118^

**Fig. 8:**
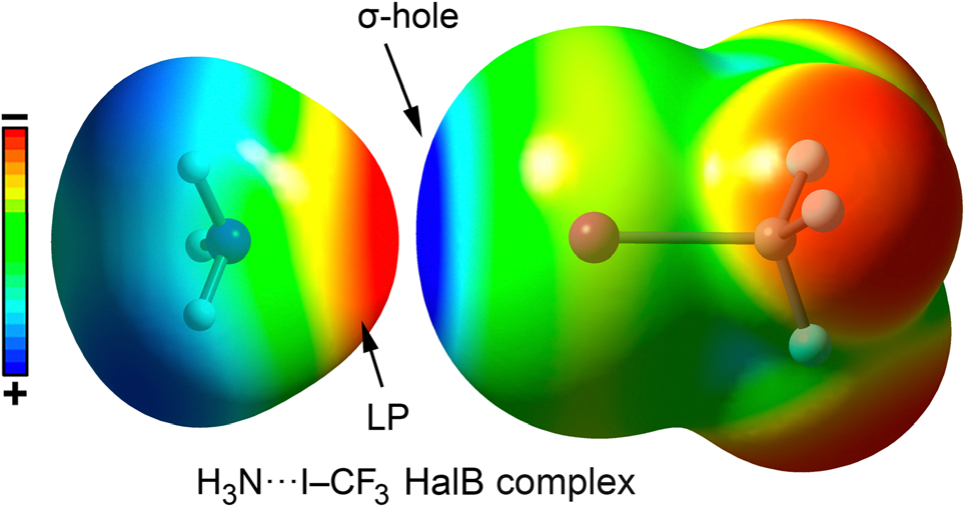
Graphical representation of a HalB complex between NH_3_ and CF_3_I using their corresponding electrostatic potential surfaces. Electropositive regions are shown in blue color and electronegative in red color.

**Fig. 9:**
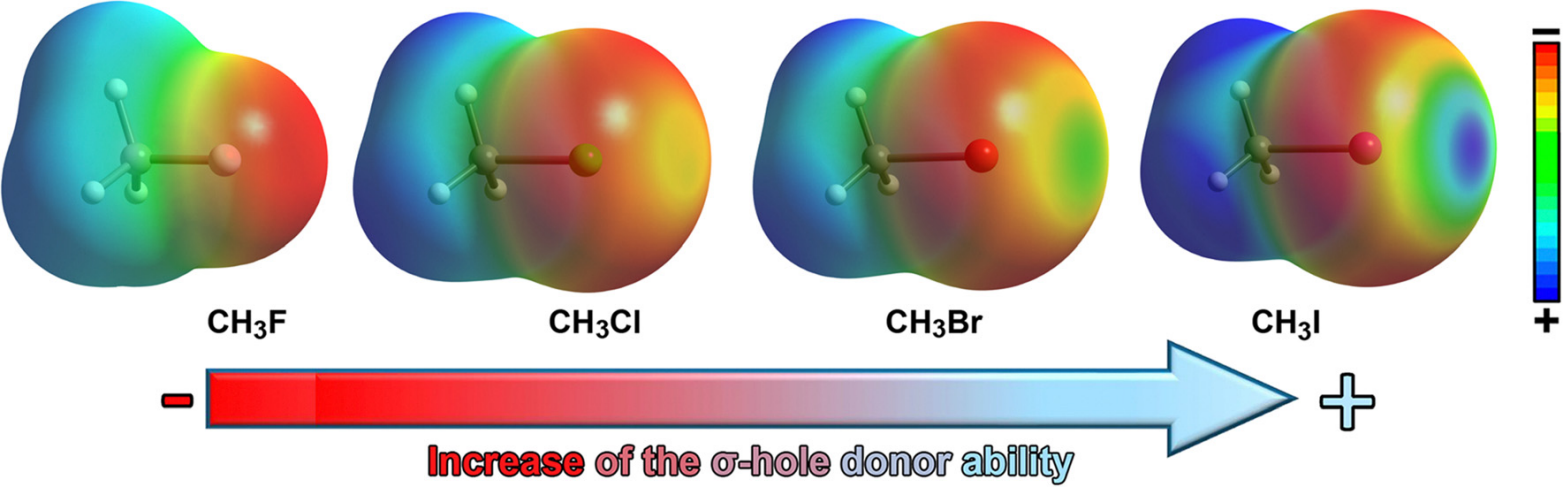
Molecular electrostatic potential (MEP) surfaces of CH_3_F, CH_3_Cl, CH_3_Br and CH_3_I. Electropositive regions are shown in blue color and electronegative in red color.

The stability of halogen bond (HalB) interactions arises from multiple contributions, including charge transfer,^119^ dispersion forces,^120^ polarization,^121^ and electrostatics.^122,123^ Typically, the electrostatic component is based on the attraction between an electron-rich molecule (or region) and a localized region of positive electrostatic potential along the extension of the Y–X bond (X = F, Cl, Br, I; Y = C), known as the σ-hole.^124^ This phenomenon is due to the polarization of halogen atoms when covalently bonded to another atom (*e.g.*, C), leading to an anisotropic electrostatic potential distribution around the halogen. Consequently, a σ-hole typically appears along the C–X bond direction, surrounded by a negatively charged σ-lump, associated with the lone electron pairs of the halogen (Fig. 8 and 9).

The σ-hole present on the halogen atom acts as an electrophilic region, facilitating favorable interactions with electron-rich species such as oxygen (O), nitrogen (N), sulfur (S), and π-systems. The polarizability of the halogen atom (which increases in the order F < Cl < Br < I) as well as the electron-withdrawing capacity of the group to which the halogen is bonded are key factors in determining the strength and directionality of the interaction. Additionally, the orbital term also contributes to the stabilization of HalBs, being usually based on the electronic donation from a lone pair or a π-system (such as a double/triple bond or an aromatic ring) belonging to the Lewis base to the σ-antibonding orbital* of the C–X bond, further strengthening the interaction.

Ho and collaborators^125–129^ have extensively studied HalB interactions in biological systems, establishing a series of criteria to consider a halogen bond as biologically relevant. These criteria are as follows:

1. It must affect the functionality of the biological system.

2. It must influence the structures and stability of biomolecules that define the system's function.

3. It must possess sufficient stability to impact biological structures and their functions.

4. It must exhibit strength comparable to that of a hydrogen bond.

Recently, our group proposed an additional criterion^130^ based on Bader's “atoms in molecules” (QTAIM) theory,^131^ consisting of characterizing the HalB interaction through a bond critical point, as outlined in the definition of this interaction proposed by the International Union of Pure and Applied Chemistry (IUPAC).^108^

Halogenated nucleotides are also incorporated into DNA or RNA to facilitate the resolution of their crystal structures using techniques such as single-wavelength anomalous dispersion (SAD) or multi-wavelength anomalous dispersion (MAD).^132,133^ These modifications can lead to minimal or localized effects as well as drastic and global structural changes,^134^ which have been studied both experimentally and theoretically.^135–140^ The following sections describe various structures in which halogen bonding plays a fundamental role in stabilizing DNA and RNA architectures.

**Fig. 10:**
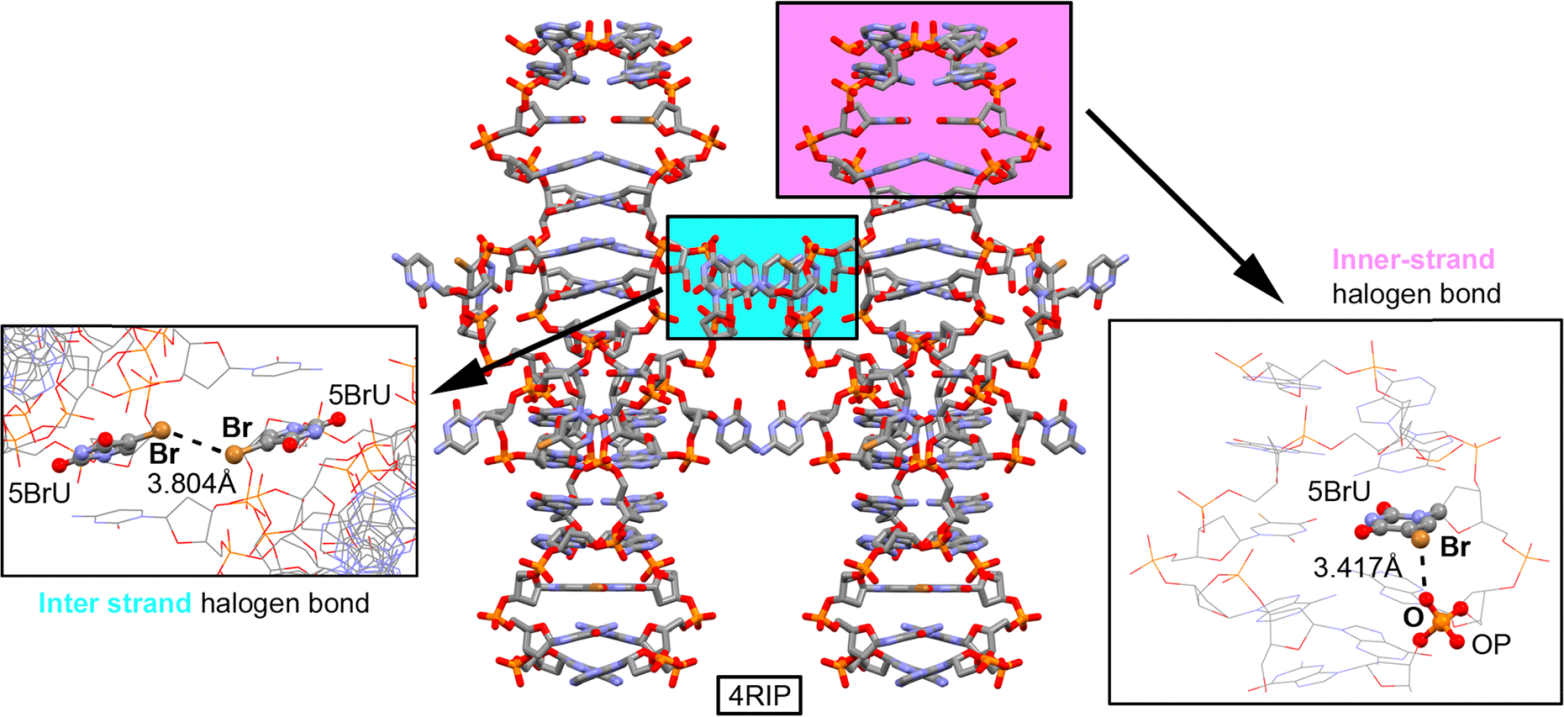
X-ray structure of a duplex corresponding to the sequence d(AC^Br^UCGGA^Br^UGA). HalB interactions are highlighted in purple and cyan and magnified within the rectangular parts of the figure.^141^

The first example is the X-ray structure of the sequence d(AC^Br^UCGGA^Br^UGA), which was used to resolve its non-brominated analog.^141^ Both sequences crystallized in a similar architecture (Fig. 10), exhibiting a duplex pattern stabilized by “head-to-head” interactions and intercalated base pairs of C⋯C, A⋯A, and G⋯G. As shown in the right side of the image, the bromine atom present in bromouridine (BrU) forms an inner-strand HalB with an oxygen atom from the phosphate group of an adjacent nucleotide, contributing to local duplex stabilization. Additionally, an inter-strand HalB is observed between two BrU nucleotides, where the bromouridine acts as both the HalB donor and the acceptor, which further stabilizes the supramolecular architecture (the left side of the image).

**Fig. 11:**
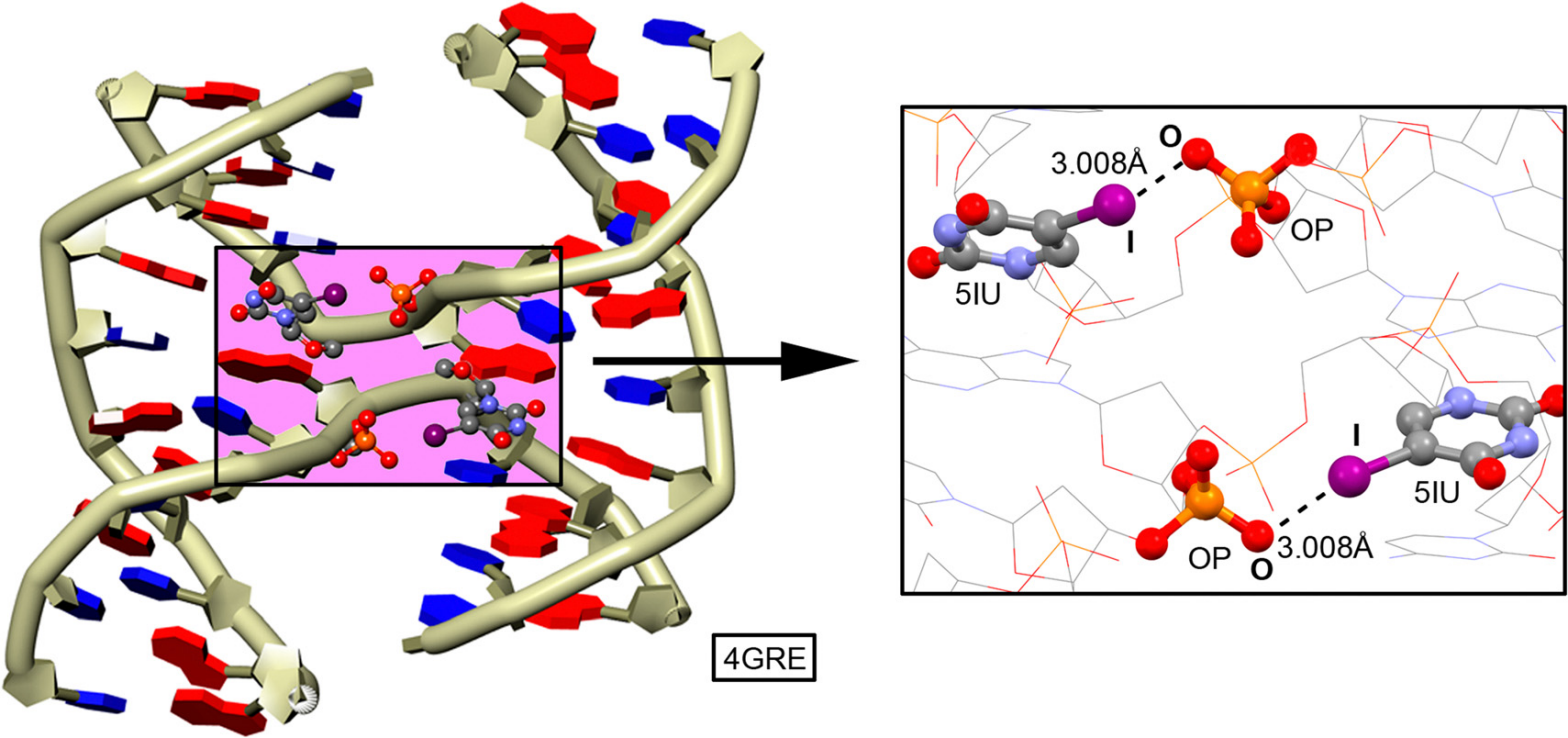
Stabilizing effect of the HalB in the formation of a Holliday junction structure.^144^ The interaction is magnified in the rectangular part of the figure.

HalB interactions have also been employed to regulate the formation of a supramolecular biological complex known as the Holliday junction,^142^ which is an intermediate structure formed by four DNA strands during cellular recombination mechanisms.^143^ To achieve this, Voth and collaborators^142^ replaced a thymine in the sequence d(CCAGTACTGG) with iodouridine (5IU), thereby facilitating the obtention of an X-ray structure (Fig. 11). The results showed that the halogenated structure corresponded to a Holliday junction, whereas the native sequence ultimately crystallized as a standard DNA duplex.^144^

Crystallographic analysis of both structures revealed HalB between the 5IU base and an oxygen atom from a neighboring phosphate group, effectively replacing the hydrogen bond typically observed in such structures.^145,146^ Subsequent competitive studies involving other halouridines (5XU, X = F, Cl, Br) demonstrated that O⋯X HalB exhibited a more stable enthalpy of formation than the hydrogen bond. This finding was further corroborated through differential scanning calorimetry (DSC) and melting studies in solution.

**Fig. 12:**
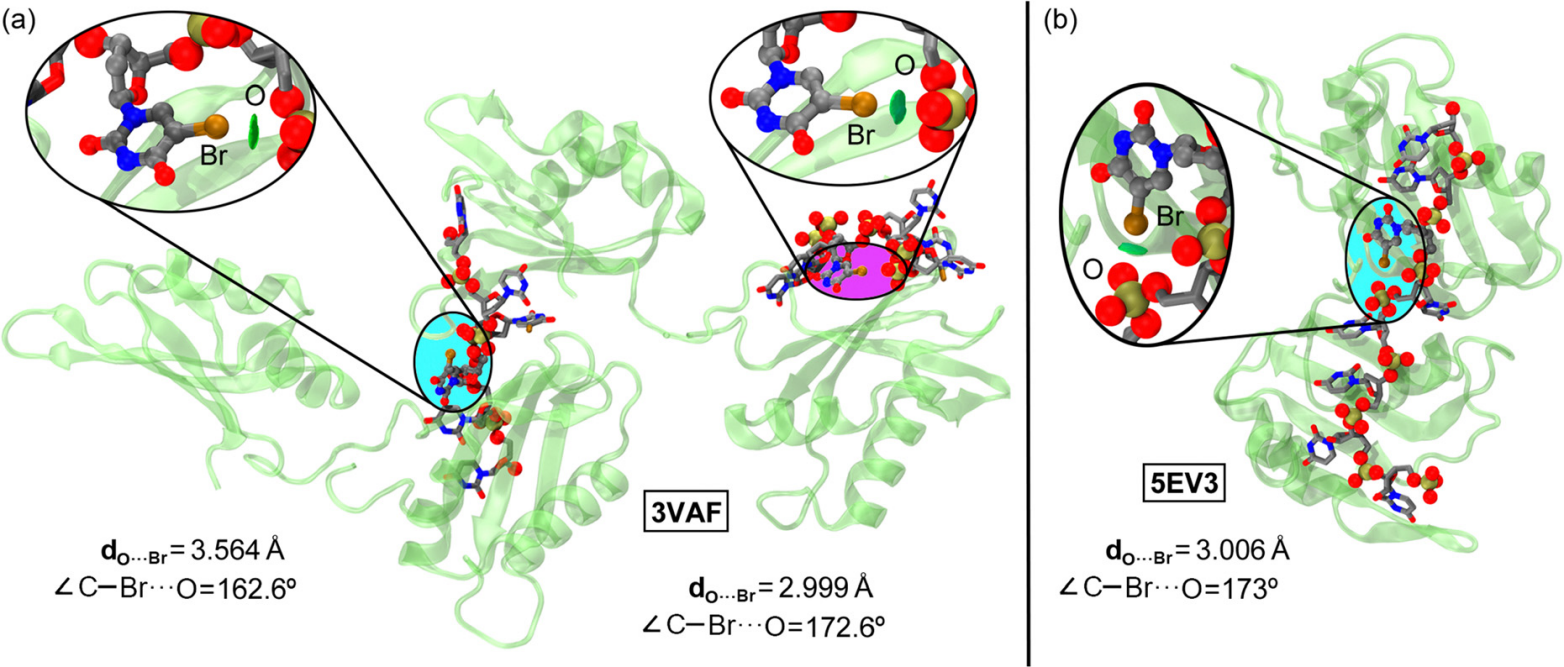
X-ray structures of 3VAF (a) and 5EV3 (b). The HalB interaction is magnified in the circular parts of the figure. The O⋯Br distances and C–Br⋯O angles are also indicated. Visualization of the halogen bond interaction using the NCIplot analysis has been included in ref. 151. Reprinted (adapted) with permission from ref. 152. Copyright 2020 American Chemical Society.

HalBs have also been observed in RNA fragments, including the MS2–RNA complex,^147^ which is considered a clear example of halogen bonding in nucleic acid structures,^148^ as well as in X-ray structures of U2AF65–RNA complexes (Fig. 12).^149,150^ In the latter, 5BrU was used to accurately determine the position of the pyrimidine base (cytosine or uracil) through X-ray diffraction. More in detail, in the 3VAF structure, the incorporation of 5BrU led to the formation of an O⋯Br halogen bond with an oxygen atom from a neighboring phosphate group, which played a crucial role in stabilizing the conformation required for protein–RNA recognition.

**Fig. 13:**
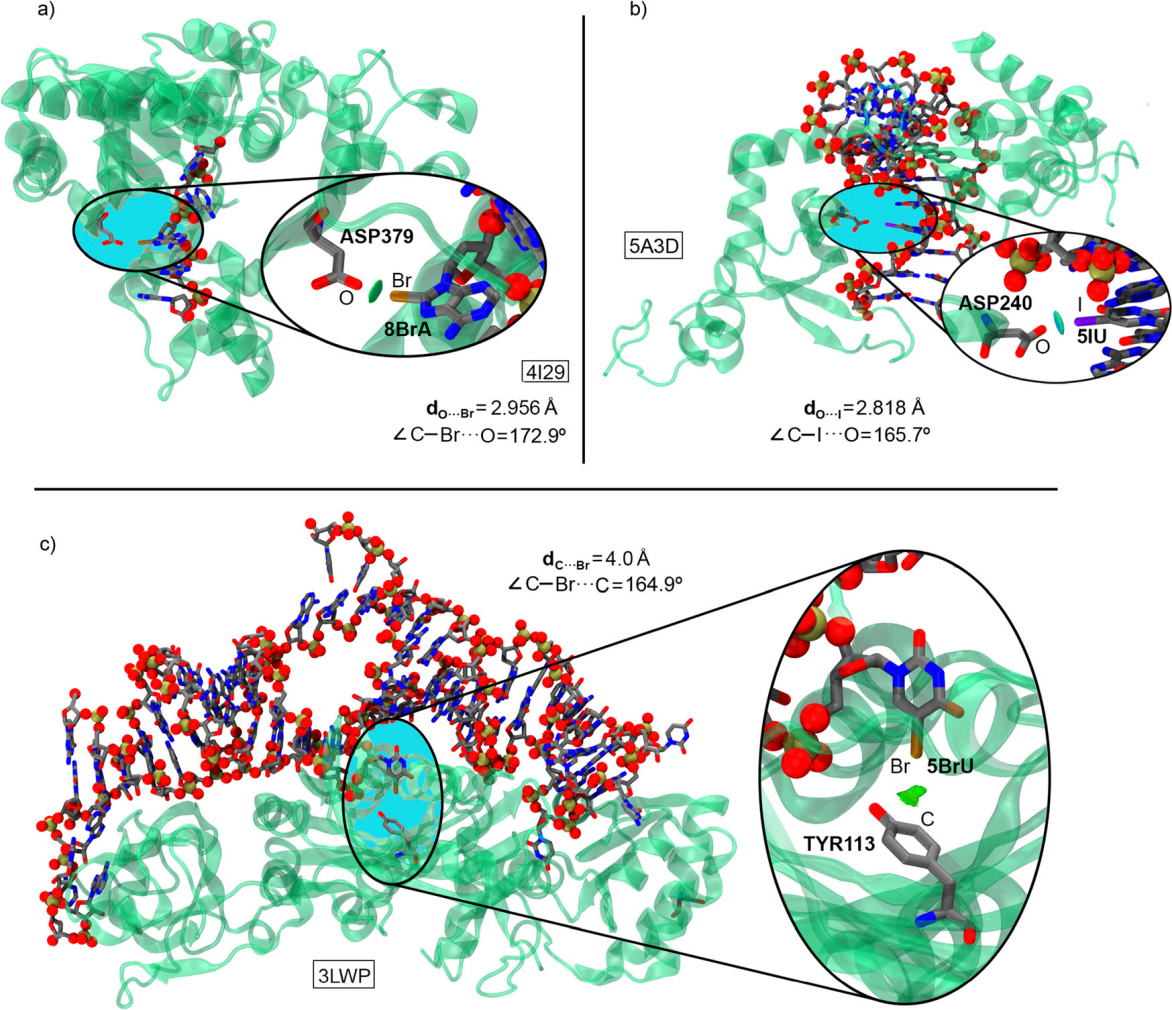
X-ray structures of 4I29 (a),^153^ 5A3D (b),^154^ and 3LWP (c).^155^ The HalB interaction is magnified in the circular parts of the figure. The O⋯Br/I distances and C–Br/I⋯O angles are also indicated. Visualization of the halogen bond interaction using the NCIplot analysis has been included in ref. 151. Reprinted (adapted) with permission from ref. 156. Copyright 2020 American Chemical Society.

Halogen bonds also play a role in protein–DNA/RNA recognition mechanisms. One example involves the Tdt protein, which belongs to the family of DNA polymerases responsible for nucleotide addition at the V(D)J union in T-cell receptors. In the study by Gouge and collaborators,^153^ a series of DNA oligomers with different lengths and compositions were described as analogs of the enzyme's pre-catalytic and post-catalytic states. To solve the 4I29 structure (Fig. 13a), an adenine was replaced by its brominated analog at the C8 position (8BrA), forming a halogen bond interaction with an oxygen atom from the carboxylate group of the ASP379 amino acid.

The second example involves the nucleotide excision repair (NER) enzyme, which belongs to the family of proteins responsible for DNA repair and serves as a primary defense against DNA damage. In this context, the study by Koch and collaborators^154^ analyzed the mechanism by which the NER protein binds to damaged DNA. In the 5A3D structure, some bases were replaced with 5-iodouracil (5IU) to facilitate crystallographic structure resolution. Two of these bases were located in the protein–DNA binding region and involved in O⋯I halogen bonds with an oxygen atom from the carboxylate group of the ASP240 amino acid (Fig. 13b), representing an additional non-covalent force contributing to protein–DNA recognition.

The final selected example involves the H/ACA RNP pseudouridine synthase enzyme, which catalyzes the isomerization of uridine to pseudouridine in RNA, a modification that plays a regulatory role in post-transcriptional RNA expression. The study by Zhou and coworkers^155^ focuses on the catalytic mechanism of pseudouridylation through the conformational analysis of the glycosidic bond of uridine in a pre-reactive state. To achieve this, the authors utilized both native RNA and RNA containing 5BrU, both labeled with ^32^P. Their findings revealed that 5BrU interacted with the protein in a way that stabilized the *anti* conformation of the nucleoside. This interaction was identified as a C⋯Br halogen bond between the bromine atom of uridine and the π-system of the aromatic amino acid TYR113 (Fig. 13c). By obtaining this non-reactive enzyme variant, the study advanced the understanding of its mechanism of action, providing an example of a biologically relevant halogen bond.

### Future perspectives

**Fig. 14:**
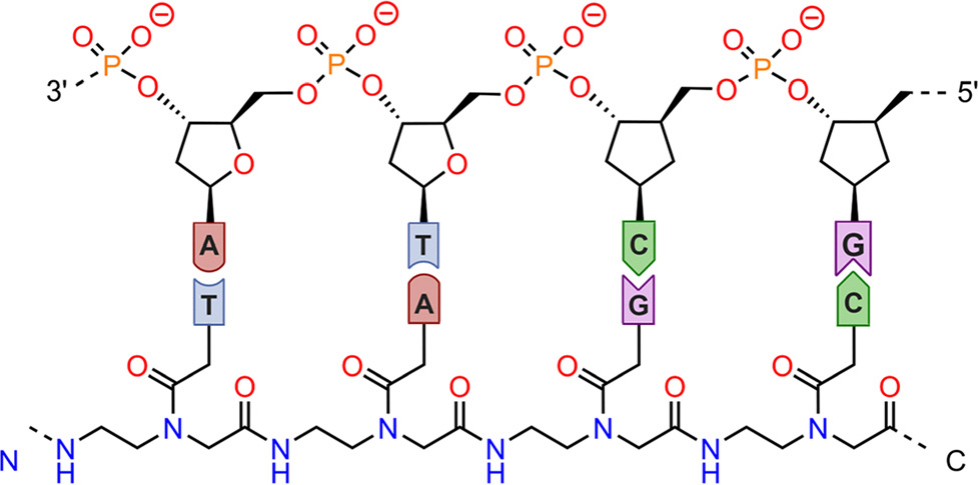
Schematic representation of a PNA–nucleic acid complex through complementary A⋯T and G⋯C pairs.

The examples shown above highlighted the impact of nucleic acid halogenation in supramolecular biology. As a future perspective, we propose the use of this non-covalent force to guide the molecular recognition of specific nucleic acid sequences, since the selective recognition of target sites in single-stranded nucleic acids (ssDNA and ssRNA) as well as in more complex structures (*e.g.* G-quadruplexes) is of crucial importance. A routine tool used in the biological area for DNA/RNA detection and sensing is peptide nucleic acids (PNAs), a family of DNA analogs first reported by Nielsen's group in 1991.^157^ In the PNA structure, the sugar-phosphate backbone of DNA is replaced by poly[N-(2-aminoethyl)glycine], while the nucleobases (A, G, C, and T) are attached to the central N atom in the monomeric unit through a –COCH_2_– linking group (Fig. 14). PNAs exhibit remarkable ability to form highly stable duplexes with complementary ssDNA and ssRNA. We believe that the incorporation of brominated/iodinated nucleobases into PNAs might trigger a specific base to base recognition motifs and form striking supramolecular DNA assemblies based on HalBs, thus paving the way for new avenues in the field of supramolecular biology. One recent example is the study by Patil and collaborators^158^ involving halogenated-U PNAs for dsRNA recognition; however, they rely on base-to-base hydrogen bonding interactions to achieve nucleic acid binding.

## Conclusions

In this review, a literature survey related to chemical modifications of nucleic acids, focusing on halogenation, has been carried out. More in detail, the structural and biological consequences of nucleobase halogenation have been discussed alongside several examples that investigate the influence of this chemical modification on the stability of nucleic acid base pairs. Furthermore, we have extended our analysis to the use of halogen bonding interactions involving brominated and iodinated nucleobases as a stabilization source of (i) novel biological supramolecular assemblies, such as “Holliday junctions”, (ii) protein conformations and (iii) protein–nucleic acid complexes. Lastly, we propose the inclusion of halogenated nucleobases into PNAs capable of forming a base to base halogen bonds as new supramolecular tools for the recognition of specific nucleic acid sequences.

## Author contributions

C. N. and J. R.-R. performed the data search and J. G.-G and A. B. designed the study and wrote the manuscript.

## Conflicts of interest

There are no conflicts to declare.

## Data Availability

No primary research results, software or code have been included and no new data were generated or analysed as part of this review.
